# Metacognitive training in patients recovering from a first psychosis: an experience sampling study testing treatment effects

**DOI:** 10.1007/s00406-017-0833-7

**Published:** 2017-08-21

**Authors:** Karin Pos, Carin J. Meijer, Oukje Verkerk, Onno Ackema, Lydia Krabbendam, Lieuwe de Haan

**Affiliations:** 1 Department of Psychiatry, Academic Medical Center (AMC), Meibergdreef 5, 1105 AZ Amsterdam, The Netherlands; 2 Early Psychosis Department, Department of Psychiatry, Amsterdam Medical Center, Amsterdam, The Netherlands; 3 Department of Educational Neuroscience, VU University Amsterdam, Amsterdam, The Netherlands; 4 LEARN! Research Institute for Learning and Education, Amsterdam, The Netherlands

**Keywords:** Early psychosis, Metacognitive training, Randomized controlled trial, Experience sampling

## Abstract

Cognitive biases, negative affect and negative self-esteem are associated with paranoia in people with psychotic disorders. Metacognitive group training (MCT) aims to target these biases although research has shown mixed results. Our objective was to establish the effect of MCT on paranoid ideation in patients with recent onset psychosis in a powerful experience sampling design. 50 patients between the age of 18 and 35 were included in a single-blind, parallel group RCT comparing MCT with occupational therapy (OT) as an active control condition. We assessed via questionnaires and experience sampling treatment effects on paranoid ideation, delusional conviction, the cognitive bias jumping to conclusion (JTC), and cognitive insight, as well as treatment effects on associations between negative affect, negative self-esteem and paranoid ideation. Patients in the MCT group did not show a decrease in paranoid ideation, delusional conviction, JTC-bias or an increase in cognitive insight compared with OT. However, negative affect showed a weaker association with paranoid ideation post-treatment in the MCT condition. In the OT condition, this association was stronger post-treatment. We tentatively suggest that patients with an early psychosis seemed to benefit from MCT in emotional learning compared with the OT condition. Despite the fact that the group training is well-received by patients, subsequent individual MCT (MCT+) may be indicated for stronger favorable effects on paranoid ideation.

## Introduction

Metacognitive training (MCT) [1] is a group intervention that aims to educate patients with psychotic disorders on cognitive and affective predictors of delusions. MCT is based on two premises, (a) cognitive biases, responsible for the erroneous processing of information, as well as negative emotions and low self-esteem, play a role in development, onset and course of psychosis [2–5], and (b) psychotic symptoms and associated distress can be alleviated by addressing underlying processes on a cognitive level [6]. The aim of MCT is to give patients knowledge on the role of cognitive biases in the rise of delusions, thereby increasing their “cognitive insight”, or the insight in the fallibility of cognitive processes. MCT focuses on different cognitive biases such as jumping to conclusions, theory of mind and attribution bias, as well as on the predictive value of depressed mood and low self-esteem on paranoid ideation. Providing patients with information on the association between delusions, cognitive biases and affective processes, as well as offering them alternative ways on how they can interpret situations, may help them develop a more nuanced belief in their “search for meaning” of situations and experiences. As a result, MCT ideally reduces paranoid ideation as well as delusional conviction concerning these paranoid beliefs. Although MCT is close to cognitive behavioral therapy in its premise that symptoms can be alleviated by addressing them on a cognitive level, it deviates from traditional CBT by focusing on cognitive biases instead of addressing core symptoms (e.g., the delusional belief). As focusing on core symptoms may be confronting and difficult for some patients, it is argued that the ‘backdoor approach’ of MCT may be easier to accept for patients than traditional CBT. One group of patients for which MCT might be particularly relevant, are people suffering from a first psychosis. Early course patterns in psychotic disorders are the strongest predictors of 15-year outcome [7]. Increasing cognitive insight and reducing cognitive biases may in turn reduce delusional conviction and consequently the risk of relapse. Thus, targeting cognitive vulnerability by means of MCT might be beneficial for long-term outcome. Research indicates that MCT is well accepted by patients in terms of treatment satisfaction and adherence [8–10]. Yet, recent meta-analyses show mixed results concerning symptomatic outcome, warranting further research on mechanisms underlying the proposed effect of MCT on delusions [11, 12]. Potential mechanisms underlying delusions, and especially paranoid ideation, have been addressed by the ‘threat anticipation model’ [2, 5]. Previous studies showed that emotions (such as depression and anxiety) and low self-esteem are associated with paranoid ideation. In addition, cognitive biases are found to predict delusions. However, studies to date have not investigated whether treatment effects of MCT may occur on the affective pathway to paranoid ideation, although the training educates patients on the interrelatedness of paranoia, mood and self-esteem. Therefore, we wanted to shed more light on the effect of MCT on both cognitive and affective pathways to paranoid ideation as explicated in the threat anticipation model. We operationalized mechanisms underlying paranoid ideation using both retrospective questionnaires and experience sampling (ESM). ESM is an ecologically valid and powerful method to study the dynamic process of person-environment interactions [13, 14]. This method addresses problems with recall- and retrospective report bias and is helpful in gaining insight in associations between daily life events, mood, delusions, cognitions and other symptoms. Common questionnaires do not provide information about these momentary associations or interactions with daily life experiences [13–15]. As it is a repeated measure design, it is a powerful tool to use in clinical studies given its sensitivity to small effects. The current study compared the effect of MCT to an active clinical control condition consisting of occupational therapy (OT). We hypothesized that compared with OT, MCT (1) would reduce paranoid ideation; (2) would reduce the cognitive bias jumping to conclusions (JTC); (3) would reduce delusion conviction; and (4) would lead to an overall increase in cognitive insight. Finally, we suspected that (5) the affective pathway towards paranoid ideation as measured with ESM would be moderated by treatment condition as MCT addresses the interrelatedness of negative affect, negative self-esteem and delusions.

## Methods

### Trial design

This study was a single-blind, parallel group randomized clinical trial conducted at the Early Psychosis Department of the Academic Medical Centre in Amsterdam, The Netherlands. It was approved by the local ethics committee under NL.42590.018.12.

### Sample

50 patients with a recent onset psychotic disorder or related disorder were included after informed consent and randomized between October 2013 and March 2015. Eligible participants were young adults (≥18 years) that recently presented themselves to the Early Psychosis Department with their first psychotic symptoms as determined by the staff based on the CASH [16], and include both in- and outpatients. Patients scoring 6–7 on one of the PANSS [17] positive items (patients suffering from serious positive symptoms that interfere substantially with their daily functioning) were excluded as this was considered a contra indication for group training. Further exclusion criteria were an IQ below 70, insufficient understanding of the Dutch language and currently receiving or ever received CBT. Subjects were randomized using http://www.randomizer.org.

### Procedure

Subjects that participated were invited for a baseline assessment. In this assessment the rationale behind the study was explained, and a written informed consent was required. Then the PANSS interview was administered to determine if the subject was eligible to participate in the study. Eligible subjects were then requested to take the tests, after which they received the Psymate ESM-palmtop with a detailed oral and written explanation on how to use it. Then for the next six consecutive days experience sampling was conducted with the Psymate. Measurement took place at baseline, and after treatment at 8 weeks. Assessors were blind to treatment allocation.

### Instruments

*Severity of psychotic symptoms at baseline* were assessed via the PANSS, a 30-item rating scale completed by clinically trained staff at the conclusion of a semi-structured interview. For this study, the positive symptoms scale (7 items) was used [18].

### Experience sampling primary and secondary clinical outcome measures

Paranoid ideation, delusion conviction, negative affect and negative self-esteem were assessed with ESM. ESM was conducted with the use of a palm computer (Psymate) with a 52 item, 7-point Likert scale questionnaire regarding appraisal of daily life events, items on different mood symptoms, negative self-esteem, hallucinations, disorganization, paranoid ideation, delusion conviction and social context at the present moment. For this study the following higher order variables were constructed: negative affect: mean score on insecure, down, lonely, anxious, irritated; negative self-esteem: mean score on “I like myself” (reversed), and “I am disappointed in myself”; and paranoid ideation: mean score on: I feel paranoid, others influence my thoughts, others are watching me, others are not what they seem. If one of the delusional experiences “others influence my thoughts”, “others are watching me”, and “others are not what they seem” were rated as 3 or higher (signifying delusional content), delusional conviction concerning the items was measured by the item “how sure are you of these experiences?”. To determine treatment effects the ESM questionnaire was scored ten times a day on six consecutive days at baseline as well as after 8 weeks of treatment. The Psymate emitted a signal at random moments between 7:30 a.m. and 10:30 p.m., after which patients filled in the questionnaires. Cronbach’s *α* was estimated on level 2 (person level) and proved to be 0.86 for paranoid ideation, 0.93 for negative affect and 0.75 for negative self-esteem.

*Paranoid thoughts and symptoms* were also measured by means of a validated Dutch version of the Green Paranoid Thought Scales (GPTS). It consists of 32 items, rated on 5-point Likert scale ranging from not at all (1) to totally (5) reflecting ideas of social reference (relevant to paranoia) and ideas of persecution, and has shown high validity and reliability in patients and healthy individuals [19].

*Cognitive insight* was measured with the Beck Cognitive Insight Scale (BCIS), a 15-item self-report scale measuring 2 constructs: the ability to acknowledge fallibility (self-reflectiveness), and certainty about belief and judgments (self-certainty). The items reflect how much the respondent agrees with each statement, using a 4-point Likert scale ranging from do not agree at all (0) to agree completely (3) The BCIS has shown to have high validity in patients [20].

*The cognitive bias* “*Jumping to conclusions*” was measured with a paper version of the ‘Beads Task’ (proportion 85/15) [21]. In this task, participants are shown two jars of coloured beads; they are informed about the relative proportions of beads in each jar. Participants are then requested, on the basis of an observed sequence, to decide on the right source of the beads. Draws to decision were calculated and JTC was defined as two or less beads needed to draw a conclusion.

### Sample size

Sample size was determined based on the known results at the time this study started (2012), indicating moderate to strong effect sizes on clinical symptoms of MCT [22] or a combined effect of MCT and MCT+ [23] in a chronic schizophrenia population. As we studied early psychosis patients, we suspected strong effects of the treatment on cognitive biases and paranoia, as biases were suspected to be more amenable than in chronic schizophrenia patients. Also we employed experience sampling, thereby increasing power to detect treatment effects on the affective pathway to paranoia. Therefore, we aimed to include 30 patients in each arm.

### Interventions

#### Metacognitive training

In the experimental condition patients received an eight sessions MCT group training (see for treatment protocol the free online course at http://clinical-neuropsychology.de/mct-psychosis-manual-dutch.html). We used the Dutch manual version (09/08) that was available at the start of this study. The training was conducted once a week by a psychiatric nurse with extensive (>5 years) experience in treating psychotic patients. Adherence to the MCT manual is enforced by its highly structured layout, thereby increasing treatment fidelity. In each session, patients were first familiarized with a particular bias via a comprehensive power-point presentation. Then, daily life examples were used to demonstrate the link between a particular cognitive bias and psychotic experiences, after which group exercises were performed with the goal to tackle these biases. Finally subjects received leaflets with information and homework assignments in the form of exercises. The goal of MCT was to teach participants about the relationship between cognitive biases and possible positive symptoms, and to motivate them to investigate these biases on a personal level. Participants were encouraged to reconsider their initial appraisals of what happens in their environment. The goal of homework assignments was to use their enhanced knowledge on biases on personal problems and to come up with alternatives for certain biases/reactions on typical, personal problems. The assumption is that when subjects become aware of the underlying processes in decisions and complexity associated with nuanced decision-making, they may change their judgment in light of this knowledge.

#### Occupational therapy

OT is a group-based therapy in which different aspects of effective functioning and skills in relation to daily life and work are addressed [24]. The occupational therapist provided information and direction to the patient with regard to any factors that will influence the recovery or development of functional abilities. To match with MCT, eight sessions were administered that centered on three different stages; (1) rapport building, (2) assessing coping strategies and strengths, and (3) facilitating the realization of long-term individual goals related to work or study. After assessment of individual goals, patients were assisted in regaining or engagement in meaningful life roles. During a typical session, all group members were working on a specified task, while focusing on their individual goals. The difficulty of the task could be adapted individually, depending on the abilities and the goals of the subject. OT tasks were chosen depending on the demands or needs of the group. Metacognitions and cognitive biases were not addressed in OT, making it an adequate active control condition. Both treatment groups received antipsychotic medication as part of TAU during the active phase of the trial.

### Data-analyses

Results were analyzed in SPSS 22. All treatment effects were analyzed on an intention to treat basis, and with use of a linear mixed model, the recommended approach to longitudinal designs as estimates are based on all available data. First, to determine treatment effect on paranoid ideation as measured with ESM, we conducted a multilevel regression analysis on paranoid ideation with treatment and period and their interaction term as predictors. As ESM data have a hierarchical structure with observations (‘beeps’) nested within subjects, beeps were assumed to be correlated within the same person as well as being correlated with adjacent beeps. Model building allowed for this correlation by nesting beeps within a subject with variance components (VC) and constraining errors in adjacent beeps by an autocorrelation structure (AR1). To determine treatment effects on delusional conviction, the abovementioned analysis was repeated.

Treatment effects on the retrospective measures of paranoid ideation, JTC-bias and cognitive insight were also measured with a linear mixed model analysis. In addition, effect sizes were calculated for treatment effects on the retrospective measures with pooled pre- and posttest SD’s [25].

Finally, to investigate whether associations between negative affect, self-esteem and paranoid ideation would be different between the two treatment groups, another linear mixed model with interaction effects of treatment × period × negative affect/negative self-esteem was conducted on ESM data, whilst defining random slopes for level 1 predictors. Both predictors were added to the same model to ensure independency of effects.

**Table 1:** Demographical and clinical information on the experimental and active control condition at baseline

Demographic information	MCT	OT	Test statistics
	Mean (SD)	Mean (SD)	
Age	23.59 (3.03)	23.08 (4.16)	*F*(1,48) = 0.25, *p* = 0.6
Sex ratio male/female	18/7	22/3	*χ* ^2^(2) = 2, *p* = 0.15
Education by levels^a^	5.28 (2.57)	5.16 (1.86)	*F*(1,48) = 0.036, *p* = 0.9
Diagnosis according to DSM IV-TR			*χ* ^2^(3) = 2.6, *p* = 0.46
Schizophrenia or schizophreniform disorder	15	15	
Psychotic disorder NOS	3	6	
Schizo-affective disorder	2	1	
Other disorder with psychotic symptoms	5	3	
Medication			
Antipsychotic medication ≥4 weeks	23	24	
Clinical variables at baseline			
GTPS paranoid ideation	47.64 (22.46)	56.16 (24.45)	*F*(1,48) = 1.51, *p* = 0.2
ESM paranoid ideation	1.8 (0.98)	2.03 (1.4)	*F*(1,2113) = 9.99, *p* = 0.002
ESM negative self-esteem	2.32 (1.17)	3 (1.45)	*F*(1,2113) = 81.3, *p* < 0.001
ESM negative affect	2.26 (1.36)	2.67 (1.36)	*F*(1,2113) = 27.26, *p* < 0.001
ESM delusional conviction when experiencing delusional content	5.08 (1.54)	5.34 (1.76)	*F*(1,737) = 2.96, *p* = 0.086
Jumping to conclusions <3 beads bias %	13 (52%)	12 (48%)	*χ* ^2^(2) = 0.08, *p* = 0.5
Self-reflectiveness (BCIS)	12.36 (3.26)	12.92 (5.86)	*F*(1,48) = 0.174, *p* = 0.7
Self-certainty (BCIS)	8.24 (3.7)	8.32 (4.06)	*F*(1,48) = 0.05, *p* = 0.9

^a^ 0 primary school, 1–3 lower vocational or secondary school, 4–5 higher secondary school, 6–8 high-school/university

## Results

**Fig. 1:**
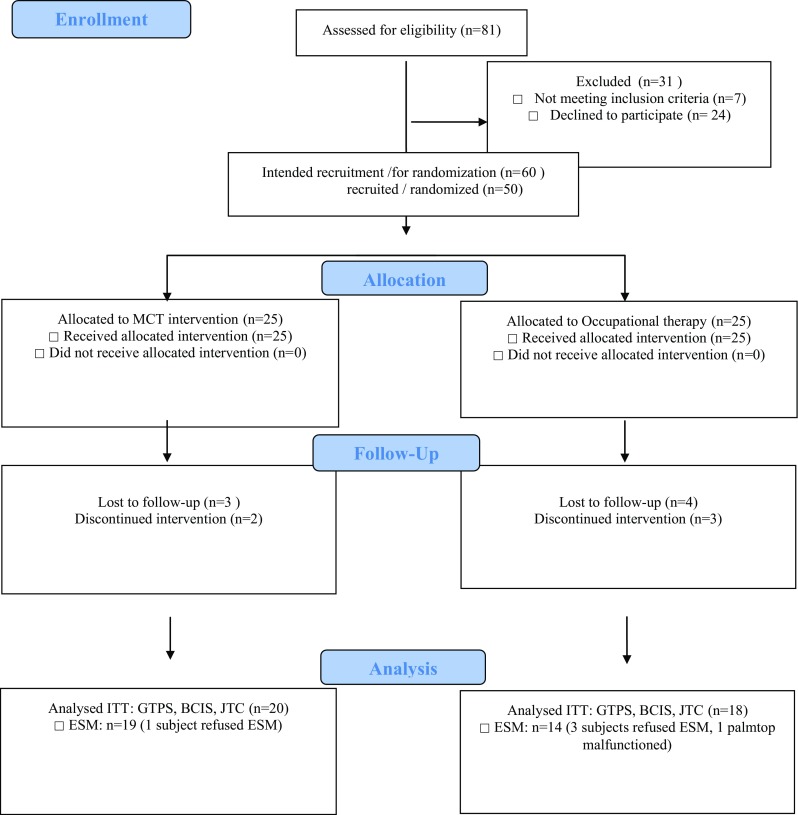
Flowchart

Figure 1 shows the flow of participants. No adverse events were reported. Table 1 shows baseline information on the study population. Groups were statistically equivalent in age, gender, education-level, and most of the clinical measures. The ESM–measures negative affect, paranoid ideation and negative self-esteem were significantly higher for the OT group compared to the MCT group at baseline. Table 2 shows the results at base-line and posttreatment for the primary outcome measures. No significant group × time interactions were found in favor of Hypotheses 1–4.

**Table 2:** Clinical variables pre- and post-treatment

Variable	Baseline		Posttreatment		Treatment × time interaction	Effect-size
	MCT	OT	MCT	OT	*p* value	
ESM	Mean (95% CI)	Mean (95% CI)	Mean (95% CI)	Mean (95% CI)		
Paranoid ideation	1.6 (1.3–2.0)	1.7 (1.4–2.1)	1.4 (1.2–1.8)	1.5 (1.2–1.8)	0.664	na
Delusion conviction	5.3 (4.6–6.1)	4.9 (4.1–5.7)	4.9 (4.2–5.7)	4.9 (4.0–5.7)	0.147	na
Retrospective questionnaires						
Paranoid ideation GPTS	47.6 (39.8–55.5)	56.2 (48.3–64.1)	35.1 (27.0–44.6)	43.4 (34.2–52.7)	0.758	0.03
Self-reflectiveness (BCIS)	12.4 (10.6–14.1)	12.9 (11.2–14.7)	13.2 (11.3–15.2)	11.4 (9.5–13.5)	0.103	0.44
Self-certainty (BCIS)	8.2 (6.8–9.7)	8.3 (6.9–9.8)	7.7 (6.2–9.3)	6.5 (4.7–8.0)	0.240	0.30
Jumping to conclusions bias % sample scoring <3	13 (52%)	12 (48%)	8 (40%)	8 (47%)	0.619	OR 1.56^a^

^a^ The chance of scoring no JTC-bias post treatment in MCT compared to pre-treatment OT

**Fig. 2:**
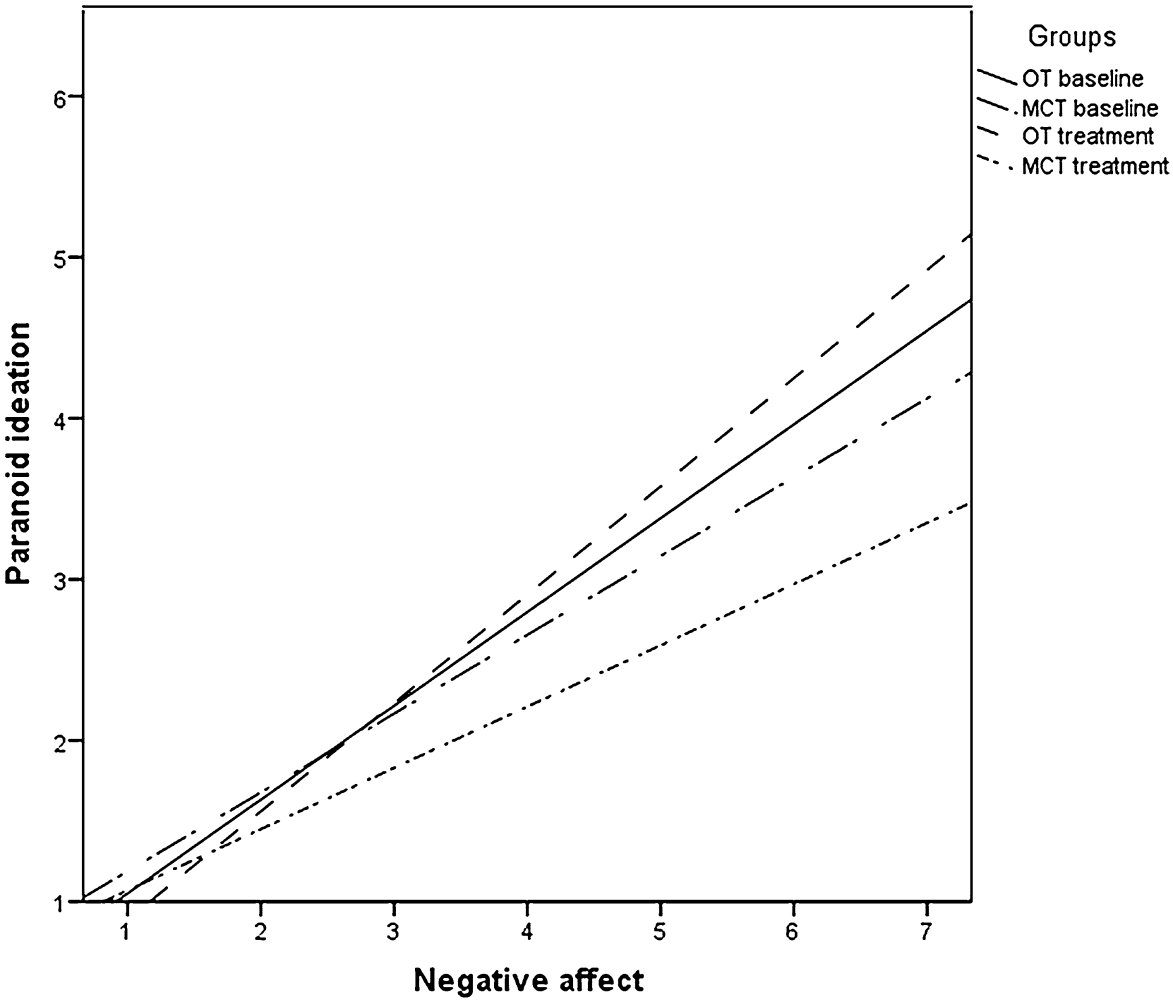
Momentary association between negative affect and paranoid ideation

A multilevel analysis showed that the interaction with time × treatment × negative self-esteem was not significant *F*(1,2113) = 0.901, *p* = 0.343. However, the interaction with time × treatment × negative affect on paranoid ideation was significant *F*(1,2113) = 16.474, *p* < 0.001, suggesting a weaker association between negative affect and paranoid ideation posttreatment for the MCT condition than for OT at baseline *B* = −0.331, 95% CI −0.442 to −0.22, *p* < 0.001, while there was a stronger association post-treatment between negative affect and paranoid ideation in the OT group *B* = 0.227, 95% CI 0.145 to 0.31, *p* < 0.001. For the MCT condition the slope between negative affect and paranoid ideation was significantly lower post-treatment compared with baseline *B* = −0.07, 95% CI −0.12 to −0.018, *p* = 0.008. To clarify this interaction, the regression lines on the original data per group were plotted in Fig. 2.

## Discussion

Our study aim was to investigate effectiveness of MCT compared to an active control condition (OT) on cognitive and affective pathways to paranoid ideation. We found no effects of MCT on paranoid ideation, JTC-bias, delusion conviction or cognitive insight compared to OT. Our study is, therefore, a replication of negative results of MCT on primary outcome data. Results from some studies in schizophrenia patients suggest that effects of MCT may be limited to heightened awareness of cognitive biases with only a modest generalization of treatment effects to actual cognitive tendencies or positive symptoms that may be related to these tendencies [8, 12, 26]. However, we conducted our study in people recovering from an early psychosis, warranting a comparison of our results within this population. Ochoa et al. [27] are to our knowledge the first to conduct an RCT on MCT in an early psychosis population. They found favorable effects on numerous cognitive biases, suggesting that this population benefits from MCT in reducing biases. Our findings further add to these results by suggesting a treatment effect on the affective route to paranoid ideation. With use of ESM we found momentary associations between negative affect and paranoid ideation that was conditional on the time × treatment interaction in favor of the MCT condition. As such, our study results suggest that our sample of early psychosis patients may have benefited from MCT in emotional learning. Next to discussing cognitive biases, MCT focusses on the predictive value of depressed mood and low self-esteem on paranoid ideation. Providing patients with information on the association between delusions and affective processes, as well as offering them alternative ways on how they can interpret situations, may have helped them develop a more accepting stance towards their affective problems, and/or a higher alertness on developing delusions in this context. Although the patients in the OT group experienced more paranoid ideation when experiencing high levels of negative affect post-treatment, these results should be interpreted in light of an overall decrease in intensity of paranoid ideation. However, it is a notable finding that mean levels of paranoid ideation in both treatment groups decreased after treatment but that patients in the MCT condition also experienced less paranoid reactivity to negative affect post treatment. We have to acknowledge the following limitations: despite randomization, the OT group scored worse on some clinical baseline ESM-measures than the MCT group and suffered higher attrition. Our results may, therefore, be partly confined by baseline differences. We studied a relatively small sample of patients; yet, the power for the ESM-analyses was ample. In addition, we have not explored the whole cycle of the threat anticipation model: most notably, we were unable to operationalize cognitive biases with ESM. Future studies should incorporate measures of the whole cycle of this model, preferably with ESM as cognitive biases may especially be prevalent in an emotional context [28]. Future studies may also investigate which bias may link negative affect to paranoid ideation as the underlying mechanism has not been clarified in our study. We operationalized JTC and cognitive insight, but other cognitive biases, such as attribution bias, may moderate or mediate the association between negative affect and paranoid ideation and may explain the treatment effects found in our study. The strengths of this study are that we were able to conduct an ESM study in a population of first episode patients. Recent meta-analyses suggest that only a few RCT’s have been conducted on MCT, whilst the training is well-received and broadly used in clinical practice. This study is conducted in light of this paucity in research findings. It is possible that the result of MCT group training is subtle and, therefore, not reflected in direct changes in paranoid ideation (on a symptom level) per se, and a first step to reduce paranoid ideation may well be a diminished association between negative affect and paranoid ideation, thus potentially breaking down a negative spiral to delusions, for which we found tentative evidence. In addition, patients were well embedded in clinical care, as such, determining treatment effects of MCT above TAU may be difficult. Alternatively, it is possible that a more focused and individualized MCT training may be necessary to further reduce paranoid ideation. Applying the newly learned skills to personal problems in daily life needs a more intensive and individual approach for which is little room in the group training [8, 29]. The group training may then serve as a positive learning experience raising cognitive awareness, and may be used as a step-up for intensive individualized CGT/MCT therapy.
